# High Fear of Discriminatory Violence among Racial, Gender, and Sexual Minority College Students and Its Association with Anxiety and Depression

**DOI:** 10.3390/ijerph19042117

**Published:** 2022-02-14

**Authors:** Erin Grinshteyn, Reid Whaley, Marie-Claude Couture

**Affiliations:** 1 Health Professions Department, School of Nursing and Health Professions, University of San Francisco, 2130 Fulton Street, San Francisco, CA 94117, USA; mcouture@usfca.edu; 2 Department of Preventive Medicine, Keck School of Medicine of USC, University of Southern California, Los Angeles, CA 90033, USA; rcwhaley@dons.usfca.edu

**Keywords:** fear, racial discrimination, gender discrimination, sexual discrimination, mental health

## Abstract

Minority students experience more discrimination on college campuses, yet little is known about fear of discrimination. This paper (a) establishes a new measure, fear of discriminatory violence, (b) assesses sociodemographic correlates of fear of discriminatory violence, and (c) estimates the effect of fear of discriminatory violence on anxiety and depression. A cross-sectional study using online surveys was undertaken among college students. A zero-inflated negative binomial model estimated the association between sociodemographics and fear of discriminatory violence. Multiple logistic regression models estimated the association between fear of discriminatory violence and anxiety/depression. Fear of discriminatory violence was higher among Black (ME: 11.9, *p* < 0.0001), Hispanic (ME: 5.9, *p* < 0.0001), Middle Eastern (ME: 5.4, *p* = 0.03), Asian (ME: 4.9, *p* < 0.0001), and multiracial (ME: 2.9, *p* < 0.0001) students compared with White students; transgender/gender non-conforming (ME: 7.2, *p* = 0.01) and female (ME: 3.4, *p* < 0.0001) students compared with male students; and gay (ME: 10.7, *p* < 0.0001), lesbian (ME: 9.0, *p* = 0.01), and bisexual students (ME: 3.4, *p* = 0.001) as well as those with a sexual orientation not included (ME: 5.5, *p* = 0.001), compared with heterosexual students. Increasing fear of discriminatory violence was associated with increased odds of anxiety (AOR: 1.04; 95% CI: 1.02, 1.06) and depression (AOR: 1.03; 95% CI: 1.02, 1.05). This understudied public health issue should be addressed to prevent fear of discriminatory violence and the resulting mental health consequences among college populations.

## 1. Introduction

Discrimination is a serious issue among college students, and racial/ethnic, gender, and sexual minority students experience discrimination at higher levels than their counterparts. Yet, little is known about fear of discriminatory violence among college students, the determinants of fear of discriminatory violence, and whether experiencing this fear is associated with mental health outcomes. We first discuss the experience of discrimination, hate crimes, hate speech, and microaggressions among racial/ethnic, gender, and sexual minority college students, and then discuss the negative mental health consequences of these experiences. We then discuss the scant research available on fear of discrimination, before briefly reviewing fear of other forms of violence and their effects on mental health among college students. While fear of discriminatory violence has yet to be studied, better understanding the experience of discrimination and other forms of fear of violence frames the need to study fear of discriminatory violence and the potential mental health consequences of this fear.

## 2. Literature Review

Studies of racial/ethnic minority college students and adolescents in the United States and other Western countries found that the majority had experienced discrimination [1,2,3]. Studies at colleges in the southwestern and northeastern United States have shown that Black students experience more perceived discrimination than other racial/ethnic groups [4,5], with one study showing that 98.5% of Black students experienced discrimination during the previous year [5]. Another study at a college in the western USA found that women of color reported more perceived discrimination than White women among a college aged sample [6]. Research at a midwestern USA university also found that gender diverse students have experienced significantly more discrimination compared with cisgender students [7]. A recent review of research in the USA and other Western countries found that sexual and gender minority groups are at increased risk of assault, harassment, bullying, and hate crimes [2].

Racial/ethnic, gender, and sexual minority college students have been shown to experience hate crimes, hate speech, and microaggressions more often than White, cis-gender, male students. Microaggressions are common, often subtle verbal, behavioral, or environmental offensive mechanisms that communicate hostile, derogatory, or negative slights or insults, and can be intentional or unintentional [8,9]. One study of Black, female students at a southeastern USA college found that almost all respondents experienced microaggressions multiple times per year [10]. Among Asian American college students, more than three quarters of the sample reported experiencing microaggressions during a two-week study period. A national study in the USA found that four out of five transgender college students reported frequently or very frequently encountering gender binary-related microaggressions [11]. Another research study using the same national sample found that almost half of gay, lesbian, bisexual, and queer college students had experienced microaggressions in the previous year [12]. Hate crimes, crimes in which the perpetrators act based on a person’s race, color, religion, or national origin [13], increased slightly on college campuses between 2010 and 2017 [14]. Over three quarters of all reported hate crimes on USA campuses were associated with the victim’s race/ethnicity, religion, or sexual orientation [14]. Still, many discriminatory crimes are not reported, leading to an underassessment.

The experience of discrimination has been associated with adverse mental health consequences. Among a national sample of LGBTQ college students in the US, microaggressions were associated with depression [15]. Microaggressions were also associated with attempted suicide among cis-gender LGBQ college students in the USA [15]. A sample from across the USA found that transgender college students reported higher rates of victimization and psychological distress compared with their cis-gender counterparts [16]. Research from a midwestern college has also shown that sexual minority college students were more likely to report mistreatment (i.e., hostility, incivility, and heterosexist harassment) on campus and that these experiences were associated with increased odds of anxiety and depression [17]. Among racial/ethnic minority adolescents in Chicago, the experience of discrimination was associated with increased risk of depression and suicidal ideation [3]. Specifically among Latino and Asian-American college students in the western US, perceived racial discrimination has been associated with increased anxiety and depression [18]. 

Less is known about fear of discriminatory violence than the experience of discrimination itself, given that this body of literature is still quite nascent. Most of the research focuses on not seeking services as a result of fear of discrimination. We define fear of discriminatory violence as fear of experiencing violent discriminatory acts such as hate crimes, hate speech, or microaggressions, all of which can be perceived as physically or emotionally abusive. Previous work has defined hate crimes as physical violence and hate speech and microaggressions as linguistic violence [19,20,21]. It has been argued by various scholars that hate speech and microaggressions are forms of linguistic violence that often lead to physical violence such as hate crimes [19,20,21]. Thus, these three concepts were aggregated together to form one measure of discriminatory violence. Some research from regional and nationally representative surveys has shown that gender and sexual minority samples delay or avoid healthcare due to fear of discrimination from their healthcare provider [22,23,24]. Nationally representative research in the USA has also shown that Black respondents compared with White respondents have avoided health care (22% versus 3%, respectively) and avoided calling the police (31% versus 2%, respectively) due to fear of discrimination [25]. Asian Americans have also been shown to avoid the doctor and the police due to fear of discrimination, according to a nationally representative sample in the USA [26]. Research in New Zealand has shown that almost half of adults with mental health conditions reported fear of discrimination due to their mental health concern [27], and a majority of those living with HIV in Serbia experienced fear of discrimination [28].

Fear of crime or violent victimization has been studied far more frequently than fear of discrimination among adolescent and college populations. Among USA samples, determinants of fear of victimization include gender [29,30,31], race/ethnicity [29,30,32], and sexual orientation [29,33]. Fear of victimization has been associated with adverse mental health outcomes among USA adolescents and college students, including increased depression, anxiety, and substance use [34,35,36].

However, little is known about fear of discriminatory violence (e.g., fear of hate crimes, fear of hate speech, fear of microaggressions) in any population. To date, we are unaware of other studies that have measured the prevalence fear of discriminatory violence and examined the sociodemographic correlates of fear of discriminatory violence among college students or the association between fear of discriminatory violence and mental health outcomes. However, given the common experience of discrimination among racial/ethnic, gender, and sexual minority groups on college campuses and the association of these experiences with adverse mental health consequences, it is possible that the *fear* of discrimination is even more prevalent than the experience, which is already highly prevalent, as the fear may be constant, and continue between experiences of discrimination. Research shows that fear of violence is often more widespread than the experience of violence. Crime in the USA has decreased over time [37]; however, fear of crime has remained steady [38]. In fact, research in Canada has even shown that being aware of violence directed toward another person within an identifiable group also leads to fear among those who are aware of the violent act [39]. Thus, it is likely that fear of discrimination is more prevalent than the experience of discrimination among members of groups that experience discrimination at high rates.

The objectives of this study are to (a) establish a new measure, fear of discriminatory violence, (b) assess the sociodemographic correlates of fear of discriminatory violence, and (c) estimate the effect of fear of discriminatory violence on (i) anxiety, and (ii) depression. It is critical to understand the determinants of fear of discriminatory violence among a diverse college sample and understand the association between fear of discriminatory violence and mental health in order to intervene in ways that can prevent fear and the resulting adverse mental health consequences among diverse college students.

## 3. Methods

### 3.1. Sample

We used a cross-sectional convenience sample of undergraduate and graduate university students at an urban university that is considered one of the most diverse in the USA [40]. Eligibility criteria included being 18 years of age or older, being a current student at the university, and being able to give informed consent. Some students (*n* = 183) aged 16 and 17 years old responded to the survey and were included in the final sample after an addendum filed with the Institutional Review Board (IRB) was approved. Recruitment emails were sent to all university students inviting them to fill out an online, self-administered, standardized questionnaire, which included questions about sociodemographic characteristics, various forms of fear, and mental health. Participants who provided consent to participate could then be entered into a raffle if they chose to provide their contact information in a separate form that was not linked to their survey data. Data were collected between October and November of 2017. This study was reviewed and approved by the university’s IRB for the Protection of Human Subjects.

### 3.2. Variables

Fear of discriminatory violence was measured using three questions about fear of hate crimes, fear of hate speech, and fear of microaggressions. Respondents were asked, “On a scale of 0 to 10 where 0 = no fear and 10 = maximum fear, how afraid are you about becoming a victim of any of these forms of victimization during the next year?” This question was asked separately for (a) hate crimes, (b) hate speech, and (c) microaggressions. These questions were adapted from previous fear of victimization measures that use similar 0 to 10 scales to ask about fear of or worry about a number of different forms of victimization [41]. The three variables were summed together to create a summary measure (range 0–30; Cronbach’s alpha = 0.90) defined as fear of discriminatory violence. A confirmatory factor analysis (CFA) was run to assess construct validity. The standardized coefficients for fear of hate crimes (coef: 0.84, *p* < 0.0001), fear of microaggressions (coef: 0.80, *p* < 0.0001), and fear of hate speech (coef: 0.97; *p* < 0.0001) were all well above suggested cutoffs, indicating that they were appropriate factor loadings for the fear of violent victimization factor. The overall model R2 was 0.95 with individual factor R2 values ranging from 0.64 to 0.94, indicating good equation level fit. This model had a high coefficient of determination (0.95), and a low standardized root mean squared residual (0.000), indicating a good model fit.

The main outcome variables in this study were anxiety and depression. Anxiety was assessed using the Generalized Anxiety Disorder (GAD-7) scale [42]. Anxiety was dichotomized classifying those with minimal and mild anxiety together (score 0–9) as “No anxiety” and those with moderate and severe anxiety (score of 10 or more) together as “Anxiety”. The Cronbach’s alpha for anxiety was 0.91. The Patient Health Questionnaire (PHQ-9) was used to assess depression [43]. This was dichotomized using previously defined cut points that classified those with no symptoms and minimal symptoms together (score 0–9) as “No Depression” and those with minor depression, major depression—moderate, and major depression—severe (score of 10 or greater) together as “Depression”. The Cronbach’s alpha for depression was 0.88. 

Race/ethnicity was measured using a comprehensive set of categories including White, Black, Hispanic, American Indian or Alaskan Native, Asian, Native Hawaiian or Pacific Islander, and Middle Eastern. Students were categorized as being one of these races/ethnicities or identified as multiracial if more than one race/ethnicity was selected. Age was measured in years and gender was self-identified as male, female, or transgender/gender nonconforming. Sexual orientation was assessed (straight, gay, lesbian, bisexual, or not included) as was citizenship status (USA citizen or not being a USA citizen). Religious affiliation included Catholic, Christian, Buddhist, Hindu, Jewish, Muslim, other faiths, or unaffiliated categories.

### 3.3. Statistical Analysis

A mean score was calculated on the fear of discriminatory violence summary measure. Bivariate analyses were conducted to compare the fear of discriminatory violence score within each potential sociodemographic correlate. Scores were compared using nonparametric tests, as they were not normally distributed. Mann–Whitney (Wilcoxon rank sum tests) and Kruskal–Wallis tests were used to compare differences in the fear of discriminatory violence variable. 

Multiple regression was used to determine the correlation between possible sociodemographic exposures and fear of discriminatory violence using a zero-inflated negative binomial model (ZINB). A zero-inflated count data model was chosen, given the large number of zeros for the fear of discriminatory violence variable in the sample (23.4%). A likelihood ratio test of alpha = 0 was run, which compared whether a zero-inflated Poisson (ZIP) or a ZINB fit the data better. This test (*p* < 0.0001) showed that the ZINB was the preferable model, as a ZINB allows for overdispersion of the outcome. Marginal effects were calculated to assess the percentage point increase or decrease in fear of discriminatory violence, with which each predictor was associated. 

Multiple logistic regression was used to examine the association between fear of discriminatory violence and the dichotomized depression and anxiety variables, controlling for a number of covariates. Covariates were selected based on the results of bivariate analyses, as well as previous literature in this area. The Hosmer–Lemeshow goodness of fit test was used to determine the model’s adequacy (*p* = 0.70 for the anxiety model and *p* = 0.44 for the depression model) and found that the models fit the data reasonably well. 

Only complete case data were included for all regression models. After assessing missingness, at most only 2% of any one predictor variable was missing. 

All regression models were tested for common method bias to determine whether a single factor confirmatory factor analysis accounted for more than half of the variance. The discriminatory violence, anxiety, and depression regressions were all well below this threshold (20.1%, 26.7%, and 26.9%, respectively) indicating that common method bias was not a concern. All variance inflation factors (VIFs) were checked as another assessment of bias, with all VIFs well below the cutoff of 3.3. All statistical analyses were performed using STATA 16.1 (Stata, College Station, TX, USA) [44].

## 4. Results

### 4.1. Sociodemographics

The mean age in this sample was 23.5 years (Table 1). The majority of the participants were female (71.8%), 26.1% were male, and 2.1% reported being transgender or gender nonconforming. This sample was racially/ethnically diverse, with 32.6% White, 28.5% Asian, 17.1% Hispanic, 14.2% multiracial, 5.5% Black, 1.5% Middle Eastern, and less than 1% Native Hawaiian/Pacific Islander. The majority of students reported their sexual orientation as straight (82.8%), 4.4% reported being gay, 1.5% reported being lesbian, 7.7% reported being bisexual, and 3.6% reported a sexual orientation that was not included. A majority of the sample were USA citizens (86.6%). Most students in this sample reported either being unaffiliated with a religion (38.9%) or Catholic (32.4%), while 15.8% reported being Christian, and much smaller proportions of the sample reported other religions or faiths. The mean score on the fear of discriminatory violence scale (Table 2) was 8.72 (SD: 8.74). These results are largely reflective of the university from which this sample was drawn, which was 63% female, 26% White, 22% Asian, 22% Hispanic, 7% multiracial, 4% Black, and 83% USA citizens at the time of data collection. No official statistics exist for the proportion of students who are Middle Eastern, Native Hawaiian/Pacific Islander, gay, lesbian, bisexual, transgender, gender nonconforming, or of various religions.

### 4.2. Bivariate Associations of Sociodemographic Correlates of Fear of Discriminatory Violence

Mean scores for fear of discriminatory violence were higher among those in the younger age categories (*p* = 0.03) (Table 3). Black students had the highest mean scores on the fear of discriminatory violence scale (mean: 15.9, SD: 10.1) followed by Middle Eastern students (mean: 13.0, SD: 10.6) and Hispanic students (mean: 11.1, SD: 9.0), while White students had the lowest mean fear of discriminatory violence scores (mean: 5.5, SD: 6.9) (*p* < 0.0001). Transgender and gender nonconforming students had the highest mean fear of discriminatory violence scores (mean: 16.9, SD: 10.0) followed by females (mean: 9.4, SD: 8.8), with males reporting the lowest fear of discriminatory violence scores (mean: 6.3, SD: 7.8) (*p* < 0.0001). Lesbian students reported the highest mean fear of discriminatory violence scores (mean: 15.4, SD: 9.3), followed by students whose sexual orientation was not included (mean: 13.8, SD: 8.9), gay (mean: 13.6, SD: 9.2), and bisexual (mean: 11.7, SD: 8.7), with heterosexual students reporting the lowest mean fear of discriminatory violence scale scores (mean: 7.9, SD: 8.5) (*p* < 0.0001). No difference in fear of discriminatory violence was observed for citizenship and religion.

### 4.3. Multiple Regression on the Sociodemographic Correlates of Fear of Discriminatory Violence

Compared with White students, Black students had an increase in fear of discriminatory violence scores of 11.9 percentage points (SE: 1.9, *p* < 0.0001), while Hispanic students had an increase of 5.9 percentage points (SE: 0.8, *p* < 0.0001), Middle Eastern students had an increase of 5.4 percentage points (SE: 2.4, *p* = 0.03), Asian students had an increase of 4.9 percentage points (SE: 0.62, *p* < 0.0001), and multiracial students had an increase of 2.9 percentage points (SE: 0.7, *p* < 0.0001) (Table 4). Transgender/gender non-conforming student reported an increase of 7.2 percentage points (SE: 2.6, *p* = 0.01) on the fear of discriminatory violence scale, and female students reported an increase of 3.4 percentage points (SE: 0.5, *p* < 0.0001), compared with male students. Compared with heterosexual students, gay students reported an increase of 10.7 percentage points (SE: 2.1, *p* < 0.0001), lesbians reported an increase of nine percentage points (SE: 3.4, *p* = 0.01), those with a sexual orientation that was not included reported an increase of 5.5 percentage points (SE: 1.7, *p* = 0.001), and bisexual students reported an increase of 3.4 percentage points (SE: 1.0, *p* = 0.001) on the fear of discriminatory violence scale. Only students reporting Jewish as their religion had a statistically significant increase in fear of discriminatory violence compared with students who reported being Catholic (ME: 6.1, SE: 2.8, *p* = 0.03). Age and citizenship status were not significantly associated with fear of discriminatory violence.

### 4.4. Multiple Logistic Regression on the Associations between Fear of Discriminatory Violence and Anxiety and Depression

With each one unit increase in the fear of discriminatory violence scale, the odds of anxiety increased by 4% (OR: 1.02, 1.06), after controlling for all other model covariates (Table 5). Similarly, with each one unit increase in the fear of discriminatory violence scale, the odds of depression increased by 3% (OR: 1.02, 1.05), after controlling for all other model covariates.

## 5. Discussion

The objectives of this study were to (a) establish a new measure, fear of discriminatory violence, (b) assess the sociodemographic correlates of fear of discriminatory violence, and (c) estimate the effect of fear of discriminatory violence on (i) anxiety, and (ii) depression. This research is the first study that we are aware of that assesses the prevalence of fear of discriminatory violence, the correlates of fear of discriminatory violence, and the association between fear of discriminatory violence and mental health outcomes among a diverse college sample. Our results show that racial, gender, and sexual minorities experienced higher levels of fear of discriminatory violence. Black, Hispanic, Asian, Middle Eastern, and multiracial students experienced significantly more fear of discriminatory violence compared with White college students. Transgender/gender nonconforming and female students reported more fear of discriminatory violence compared with male students. Sexual orientation was also significantly associated with fear of discriminatory violence, with gay students reporting the highest scores followed by lesbian students, students with an orientation not included, and bisexual students, compared with heterosexual students. Age, however, was not significantly associated with fear of discriminatory violence after controlling for other factors. It is possible that because the majority of respondents in this study were younger than 30 years of age, due to the sample being drawn from a college, there was less variability and less of an effect of age. Younger people have shown less fear when studying fear of crime; thus, it is possible that due to this sample being younger, fear associated with age was less detectable. Citizenship status was also not significantly associated with fear of discriminatory violence. While, initially, it may seem that this measure should be associated with fear of discriminatory violence, it may be that the measure of citizenship is too broad to detect meaningful differences. Those included in the “non-citizen” category include those with green cards and student visas, who may not experience fear of discriminatory violence the way students with an undocumented legal status may experience this fear. Finally, fear of discriminatory violence was significantly associated with increased depression and anxiety. While the effect size for a one unit increase in fear of discriminatory violence was on the smaller end (4% for anxiety and 3% for depression), it is still meaningful. Stated another way, a ten percent increase in fear of discriminatory violence would be associated with a 12% increase in anxiety and a 9% increase in depression. A one standard deviation increase in discriminatory violence was associated with a 34.8% increase in anxiety and 26.1% increase in depression. It is crucial that we better understand fear of discriminatory violence and their higher prevalence among racial/ethnic, gender, and sexual minority groups, as understanding these disparities is necessary to better target prevention efforts and interventions.

Our results support findings from previous research, showing that fear of different forms of victimization is higher in minority populations, while adding to the literature by showing that fear of this newly defined form of violence is higher among the same groups. Fear of violence among transgender and gender nonconforming students is significantly higher than among their cis-gender counterparts [29]. Racial/ethnic minority college students experience more fear of violence [29] and more fear of bullying [36], compared with white students. This research finds similar patterns for fear of discriminatory violence. Though little literature has assessed fear of discriminatory violence, a large number of studies have shown that racial/ethnic, gender, and sexual minorities experience far more discrimination than their White, cis-gender, heterosexual counterparts [1,4,5,6,7,45]. Our findings show a similar pattern with the same groups reporting greater fear of discriminatory violence, which has not previously been studied. In addition, previous research has established that fear of violent victimization has been associated with depression and anxiety [31,35] among adolescents, and substance use among college students [34]. These findings are aligned with our research showing that fear of discriminatory violence is also associated with increased anxiety and depression among a diverse college sample. 

### 5.1. Limitations

This study used a convenience sample; thus, those who responded to the survey may not be representative of the general population of university students. However, the sample was fairly representative of the population of students of the university from which it was drawn. While these analyses are more representative of fear of discriminatory violence among racial/ethnic, gender, and sexual minority populations, which was the intent of this research, it may not be representative of all university students. The study design was cross-sectional, thus making causality impossible to establish. While this new measure of fear of discriminatory violence was adapted from existing measures of fear of crime that measure analogous concepts on a scale from zero to ten and was validated in this sample using the reliability coefficient (Cronbach’s alpha) and a confirmatory factor analysis, both of which provided support for this new measure of discriminatory violence, this measure has not been validated in a representative sample. The information collected was self-reported, which could lead to recall and social desirability biases. While there was very little missing data, and largely there were no differences between those with and without missing data, unadjusted tests showed that those with missing data on race/ethnicity had lower depression and anxiety scores than those without missing data for race/ethnicity. This may slightly overestimate depression and anxiety scores, but only very slightly, as only 2% of all respondents had missing data for race/ethnicity. Finally, residual confounding by unmeasured confounders is possible. 

### 5.2. Implications

More research needs to be completed to better understand fear of discriminatory violence and its consequences. Compared with fear of crime, fear of discriminatory violence is a newer measurement, and thus more research should use this to validate and assess this measure among larger and more representative samples. Research should continue to understand the determinants and consequences of fear of discriminatory violence among college students who are at greater risk of this fear. Longitudinal studies should be undertaken to better assess the temporal nature between the determinants of fear of discriminatory violence and the effects of fear on mental health. 

Colleges and universities should consider how experiencing fear of discriminatory violence affects their racial/ethnic, sexual, and gender minority students, and address this issue using existing university infrastructure (e.g., mental health services, health services, offices of diversity, cultural centers, student services, student life, housing, student organizations). Leveraging these existing stakeholders and university infrastructure to address fear of discriminatory violence will be required to assemble a multilevel response that appropriately and adequately addresses fear of discriminatory violence among these different minority groups. While the university from which this sample was drawn does have existing student centers on campus that serve to support students through on campus programming related to race, ethnicity, class, gender, and sexuality, as well as many student clubs for a variety of sociodemographic groups, it is likely that these services should be expanded and improved upon both in terms of what currently exists and how much operational support they receive. 

A brief screening tool could be developed and tested among college samples that would assess fear of discriminatory violence and identify at-risk students. This type of tool, which has been effective in screening for mental health concerns among college students [46,47,48], could help providers assess fear of discriminatory violence among students, which could be an indicator of possible mental health concerns, as well. Identifying students who are at risk of mental health issues earlier, by screening for fear of discriminatory violence, would lead to better outcomes both in fear reduction and in anxiety and depression management. 

Numerous interventions aimed at reducing the perpetration of discrimination have been developed and assessed among adolescents and college students. While these have not been evaluated for their affect on fear of discriminatory violence, they have been assessed for their affect on actual discrimination. Gay–straight alliances and anti-homophobic bullying policies have been associated with reduced discrimination [49] and should be evaluated to determine whether they also reduce fear of discriminatory violence. Research has shown that talking about race may reduce prejudice among students [50], and research examining trainings found that those trained on stereotype replacement, counter-stereotypic imaging, individuation, perspective talking, and increasing opportunities for contact saw increases in concern about discrimination and awareness of bias [51]. Future evaluations of those programs should also assess whether they reduce fear of discriminatory violence among participants, especially among various racial/ethnic, gender, and sexual minority students. 

## 6. Conclusions

Universities and colleges must take fear of discriminatory violence seriously among their student populations, especially racial/ethnic, gender, and sexual minority students. Using existing school infrastructure to develop and implement interventions could result in effective reductions in fear of discriminatory violence among college students, as well as resulting anxiety and depression. Fear of discriminatory violence is still a very new area of research, and more will need to be done to better understand this serious, consequential public health issue.

## Figures and Tables

**Table 1:** Demographic characteristics of university students (*n* = 1415).

Characteristic	*n* (%)	Mean (SD)
Age	--	23.46 (7.40)
Race/Ethnicity		
White	451 (32.56)	
Black	76 (5.49)	
Hispanic	237 (17.11)	
AIAN	0 (0.00)	
Asian	395 (28.52)	
NH/PI ^a^	9 (0.65)	
Middle Eastern	21 (1.52)	
Multiracial	196 (14.15)	
Gender		
Male	368 (26.10)	
Female	1013 (71.84)	
Transgender/GNC ^b^	29 (2.06)	
Sexual Orientation		
Straight	1168 (82.83)	
Gay	62 (4.38)	
Lesbian	21 (1.49)	
Bisexual	109 (7.73)	
Not Included	50 (3.55)	
USA Citizen		
Yes	1225 (86.57)	
No	190 (13.42)	
Religious Preference		
Christian: Catholic	432 (32.43)	
Christian: Other	211 (15.84)	
Buddhist	48 (3.60)	
Hindu	19 (1.43)	
Jewish	25 (1.88)	
Muslim	37 (2.78)	
Other faiths	42 (3.15)	
Unaffiliated	518 (38.89)	

^a^ Native Hawaiian/Pacific Islander, ^b^ Gender non-conforming.

**Table 2:** Mean fear of discriminatory violence scores among university students (*n* = 1415).

Fear Outcome	Mean (SD)
Scale (0–10):	
Fear of microaggressions	3.40 (3.37)
Fear of hate crimes	2.57 (3.09)
Fear of hate speech	2.79 (3.12)
Scale (0–30):	
Total fear of discriminatory violence	8.72 (8.74)

**Table 3:** Bivariate associations between sociodemographic factors and fear of discriminatory violence among university students (*n* = 1415).

Characteristic	Fear of Discriminatory Violence Mean (SD)
Age	
16–20 years	**8.34 (8.52)**
21–25 years	**9.68 (9.31)**
26–30 years	**9.33 (8.39)**
>30 years	**7.62 (8.61)**
Race/Ethnicity	
White	**5.52 (6.85)**
Black	**15.89 (10.09)**
Hispanic	**11.12 (8.95)**
Asian	**9.64 (8.93)**
NH/PI ^a^	**9.00 (9.44)**
Middle Eastern	**12.95 (10.59)**
Multiracial	**7.99 (8.18)**
Gender	
Male	**6.32 (7.77)**
Female	**9.35 (8.78)**
Trans/GNC ^b^	**16.90 (10.04)**
Sexual Orientation	
Straight	**7.86 (8.45)**
Gay	**13.61 (9.17)**
Lesbian	**15.40 (9.31)**
Bisexual	**11.72 (8.70)**
Not Included	**13.77 (8.93)**
USA Citizen	
Yes	8.63 (8.64)
No	9.31 (9.39)
Religious Preference	
Christian: Catholic	8.99 (9.01)
Christian: Other	8.56 (8.56)
Buddhist	10.44 (8.39)
Hindu	6.42 (7.58)
Jewish	9.4 (8.06)
Muslim	11.89 (10.48)
Other faiths	9.19 (9.53)
Unaffiliated	8.14 (8.38)

^a^ Native Hawaiian/Pacific Islander, ^b^ Gender non-conforming. Bolded results are statistically significant at the 0.05 level.

**Table 4:** Marginal effects examining the association between sociodemographic factors and fear of discriminatory violence among university students (*n* = 1415).

Characteristic	Marginal Effect (SE)	(95% CI)
Age	0.02 (0.03)	(−0.05, 0.09)
Race/Ethnicity		
White (ref)	--	--
Black	**11.94 (1.92)**	**(8.18, 15.70)**
Hispanic	**5.93 (0.79)**	**(4.38, 7.48)**
Asian	**4.87 (0.62)**	**(3.66, 6.07)**
NH/PI ^a^	3.93 (3.16)	(−2.26, 10.12)
Middle Eastern	**5.35 (2.41)**	**(0.62, 10.08)**
Multiracial	**2.93 (0.69)**	**(1.58, 4.29)**
Gender		
Male (ref)	--	--
Female	**3.75 (0.51)**	**(2.74, 4.75)**
Trans/GNC ^b^	**7.21 (2.60)**	**(2.11, 12.30)**
Sexual Orientation		
Straight (ref)	--	--
Gay	**10.66 (2.14)**	**(6.46, 14.85)**
Lesbian	**9.00 (3.40)**	**(2.34, 15.66)**
Bisexual	**3.42 (1.00)**	**(1.45, 5.39)**
Not Included	**5.45 (1.71)**	**(2.11, 8.79)**
USA Citizen		
Yes (ref)	--	--
No	1.01 (0.84)	(−0.63, 2.65)
Religious Preference		
Christian: Catholic (ref)	--	--
Christian: Other	−1.35 (0.73)	(−2.78, 0.09)
Buddhist	0.76 (1.36)	(−1.91, 3.43)
Hindu	−2.63 (1.61)	(−5.79, 0.53)
Jewish	**6.07 (2.79)**	**(0.60, 11.54)**
Muslim	3.06 (2.20)	(−1.25, 7.38)
Other faiths	0.57 (1.61)	(−2.59, 3.74)
Unaffiliated	−0.37 (0.61)	(−1.56, 0.82)

^a^ Native Hawaiian/Pacific Islander, ^b^ Gender non-conforming. Bolded results are statistically significant at the 0.05 level.

**Table 5:** Multiple logistic regression models estimating the association between fear of discriminatory violence and anxiety and depression among university students (*n* = 1415).

	Anxiety		Depression	
Characteristic	Odds Ratio (SE)	(95% CI)	Odds Ratio (SE)	(95% CI)
Fear of Discriminatory Violence	**1.04 (0.01)**	**(1.02, 1.06)**	**1.03 (0.01)**	**(1.02, 1.05)**
Age	0.98 (0.01)	(0.96, 1.00)	**0.97 (0.01)**	**(0.95, 0.99)**
Race/Ethnicity				
White (ref)	--	--	--	--
Black	0.63 (0.21)	(0.32, 1.23)	1.66 (0.48)	(0.94, 2.92)
Hispanic	0.91 (0.19)	(0.61, 1.37)	1.23 (0.23)	(0.85, 1.78)
Asian	0.72 (0.13)	(0.50, 1.04)	1.03 (0.17)	(0.74, 1.43)
NH/PI ^a^	1.09 (0.93)	(0.20, 5.80)	1.71 (1.30)	(0.39, 7.57)
Middle Eastern	**3.05 (1.54)**	**(1.14, 8.18)**	**3.07 (1.53)**	**(1.16, 8.14)**
Multiracial	1.27 (0.26)	(0.85, 1.91)	1.39 (0.27)	(0.95, 2.04)
Gender				
Male (ref)	--	--	--	
Female	1.30 (0.23)	(0.92, 1.84)	1.03 (0.16)	(0.76, 1.39)
Trans/GNC ^b^	2.14 (0.95)	(0.89, 5.12)	**2.46 (1.12)**	**(1.01, 6.00)**
Sexual Orientation				
Straight (ref)	--	--	--	
Gay	1.36 (0.45)	(0.71, 2.61)	0.92 (0.29)	(0.49, 1.72)
Lesbian	2.18 (1.07)	(0.84, 5.69)	1.99 (0.97)	(0.76, 5.17)
Bisexual	**1.67 (0.39)**	**(1.06, 2.63)**	**1.79 (0.39)**	**(1.16, 2.76)**
Not Included	2.11 (0.08)	(0.14, 0.49)	1.48 (0.50)	(0.77, 2.86)

^a^ Native Hawaiian/Pacific Islander, ^b^ Gender non-conforming. Bolded results are statistically significant at the 0.05 level.

## Data Availability

The data presented in this study may be available on request from the corresponding author. The data are not publicly available due to the fact that the authors are still analyzing the data.

## References

[1] Juang L., Ittel A., Hoferichter F., Gallarin M.M. (2016). Perceived Racial/Ethnic Discrimination and Adjustment among Ethnically Diverse College Students: Family and Peer Support as Protective Factors. J. Coll. Stud. Dev..

[2] McKay T., Lindquist C.H., Misra S. (2019). Understanding (and Acting On) 20 Years of Research on Violence and LGBTQ + Communities. Trauma Violence Abus..

[3] Tobler A.L., Maldonado-Molina M.M., Staras S.A.S., O’Mara R.J., Livingston M.D., Komro K.A. (2013). Perceived racial/ethnic discrimination, problem behaviors, and mental health among minority urban youth. Ethn. Health.

[4] Cokley K., Smith L., Bernard D., Hurst A., Jackson S., Stone S., Awosogba O., Saucer C., Bailey M., Roberts D. (2017). Impostor feelings as a moderator and mediator of the relationship between perceived discrimination and mental health among racial/ethnic minority college students. J. Couns. Psychol..

[5] Prelow H.M., Mosher C.E., Bowman M.A. (2006). Perceived Racial Discrimination, Social Support, and Psychological Adjustment among African American College Students. J. Black Psychol..

[6] Daftary A.-M., Devereux P., Elliott M. (2020). Discrimination, depression, and anxiety among college women in the Trump era. J. Gend. Stud..

[7] Thompson A.E., Shortreed R.L., Moore E.A., Carey-Butler S.R. (2019). Gender diverse college students’ perceptions of climate and discriminatory experiences. J. LGBT Youth.

[8] Pierce C. (1970). Offensive Mechanisms. Black Seventies.

[9] Sue D.W., Capodilupo C.M., Torino G.C., Bucceri J.M., Holder A., Nadal K.L., Esquilin M. (2007). Racial microaggressions in everyday life: Implications for clinical practice. Am. Psychol..

[10] Donovan R.A., Galban D.J., Grace R.K., Bennett J.K., Felicié S.Z. (2013). Impact of Racial Macro- and Microaggressions in Black Women’s Lives: A Preliminary Analysis. J. Black Psychol..

[11] Woodford M.R., Joslin J.Y., Pitcher E.N., Renn K.A. (2017). A Mixed-Methods Inquiry into Trans* Environmental Microaggressions on College Campuses: Experiences and Outcomes. J. Ethn. Cult. Divers. Soc. Work.

[12] Ylioja T., Cochran G., Woodford M.R., Renn K.A. (2018). Frequent Experience of LGBQ Microaggression on Campus Associated with Smoking Among Sexual Minority College Students. Nicotine Tob. Res..

[13] FBI Hate Crimes. What We Investigate. https://www.fbi.gov/investigate/civil-rights/hate-crimes.

[14] National Center for Education Statistics Indicator 22: Hate Crime Incidents at Postsecondary Institutions. Indicators of School Crime and Safety, July 2020. https://nces.ed.gov/programs/crimeindicators/ind_22.asp.

[15] Woodford M.R., Weber G., Nicolazzo Z., Hunt R., Kulick A., Coleman T., Coulombe S., Renn K.A. (2018). Depression and Attempted Suicide among LGBTQ College Students: Fostering Resilience to the Effects of Heterosexism and Cisgenderism on Campus. J. Coll. Stud. Dev..

[16] Effrig J.C., Bieschke K.J., Locke B.D. (2011). Examining Victimization and Psychological Distress in Transgender College Students. J. Coll. Couns..

[17] Woodford M.R., Han Y., Craig S., Lim C., Matney M.M. (2014). Discrimination and Mental Health among Sexual Minority College Students: The Type and Form of Discrimination Does Matter. J. Gay Lesbian Ment. Health.

[18] Hwang W.-C., Goto S. (2008). The impact of perceived racial discrimination on the mental health of Asian American and Latino college students. Cult. Divers. Ethn. Minority Psychol..

[19] Matsuda M. (1988). Public response to racist speech: Considering the victim’s story. Mich. Law Rev..

[20] Tirrell L. (2012). Genocidal language games. Speech and Harm: Controversies over Free Speech.

[21] McClure E. (2020). Escalating Linguistic Violence from Microaggressions to Hate Speech. Microaggressions and Philosophy.

[22] Casey L.S., Reisner S.L., Findling M.G., Blendon R.J., Benson J.M., Sayde J.M., Miller C. (2019). Discrimination in the United States: Experiences of lesbian, gay, bisexual, transgender, and queer Americans. Health Serv. Res..

[23] Glick J.L., Theall K.P., Andrinopoulos K.M., Kendall C. (2018). The Role of Discrimination in Care Postponement among Trans-Feminine Individuals in the U.S. National Transgender Discrimination Survey. LGBT Health.

[24] Seelman K.L., Colón-Diaz M.J.P., LeCroix R.H., Xavier-Brier M., Kattari L. (2017). Transgender Noninclusive Healthcare and Delaying Care Because of Fear: Connections to General Health and Mental Health Among Transgender Adults. Transgender Health.

[25] Bleich S.N., Findling M.G., Casey L.S., Blendon R.J., Benson J.M., SteelFisher G.K., Sayde J.M., Miller C. (2019). Discrimination in the United States: Experiences of black Americans. Health Serv. Res..

[26] McMurtry C.L., Findling M.G., Casey L.S., Blendon R.J., Benson J.M., Sayde J.M., Miller C. (2019). Discrimination in the United States: Experiences of Asian Americans. Health Serv. Res..

[27] Peterson D., Pere L., Sheehan N., Surgenor G. (2006). Experiences of mental health discrimination in New Zealand. Health Soc. Care Community.

[28] Baros S., Bozinovic-Knezevic A. (2020). Experiences of discrimination among people living with HIV in Serbia. Eur. J. Public Health.

[29] Grinshteyn E., Whaley R., Couture M.-C. (2020). Minority Report: Prevalence of Fear of Violent and Property Crimes Among a Diverse College Sample. Soc. Indic. Res..

[30] Grinshteyn E.G., Eisenman D.P., Cunningham W.E., Andersen R., Ettner S.L. (2016). Individual-and neighborhood-level determinants of fear of violent crime among adolescents. Fam. Community Health.

[31] Lee D., Hilinski-Rosick C. (2011). The Role of Lifestyle and Personal Characteristics on Fear of Victimization among University Students. Am. J. Crim. Justice.

[32] May D.C., Dunaway R.G. (2000). Predictors of fear of criminal victimization at school among adolescents. Sociol. Spectr..

[33] Meyer D., Grollman E.A. (2014). Sexual Orientation and Fear at Night: Gender Differences among Sexual Minorities and Heterosexuals. J. Homosex..

[34] Couture M.-C., Garcia D., Whaley R., Grinshteyn E. (2020). Effect of fear of victimization on hazardous alcohol drinking, tobacco, and marijuana use among university students: A tale of two sexes. Addict. Behav..

[35] Grinshteyn E.G., Cunningham W.E., Eisenman D.P., Andersen R., Ettner S.L. (2017). Fear of violent crime and anxiety/depression among adolescents. Ment. Health Prev..

[36] Grinshteyn E.G., Whaley R., Couture M.-C. (2021). Fear of Bullying and Its Effects on Mental Health among College Students: An Emerging Public Health Issue. J. Sch. Violence.

[37] Gramlich J. (2016). Voters’ Perceptions of Crime Continue to Conflict with Reality. Pew Research Center. https://www.pewresearch.org/fact-tank/2016/11/16/voters-perceptions-of-crime-continue-to-conflict-with-reality/.

[38] (2019). Gallup, Crime. https://news.gallup.com/poll/1603/Crime.aspx.

[39] Perry B., Alvi S. (2012). ‘We are all vulnerable’: The *in terrorem* effects of hate crimes. Int. Rev. Vict..

[40] U.S. News & World Report Campus Ethnic Diversity. https://www.usnews.com/best-colleges/rankings/national-universities/campus-ethnic-diversity.

[41] Williams F.P., McShane M.D., Akers R.L. (2000). Worry about victimization: An alternative and reliable measure for fear of crime. West. Criminol. Rev..

[42] Spitzer R.L., Kroenke K., Williams J.B.W., Löwe B. (2006). A Brief Measure for Assessing Generalized Anxiety Disorder: The GAD-7. Arch. Intern. Med..

[43] Kroenke K., Spitzer R.L. (2002). The PHQ-9: A New Depression Diagnostic and Severity Measure. Psychiatr. Ann..

[44] StataCorp (2017). Stata: Release 15. College Station.

[45] Dugan J.P., Kusel M.L., Simounet D.M. (2012). Transgender College Students: An Exploratory Study of Perceptions, Engagement, and Educational Outcomes. J. Coll. Stud. Dev..

[46] DeMartini K.S., Carey K.B. (2012). Optimizing the use of the AUDIT for alcohol screening in college students. Psychol. Assess..

[47] Khubchandani J., Brey R., Kotecki J., Kleinfelder J., Anderson J. (2016). The Psychometric Properties of PHQ-4 Depression and Anxiety Screening Scale among College Students. Arch. Psychiatr. Nurs..

[48] Seigers D.K.L., Carey K.B. (2010). Screening and Brief Interventions for Alcohol Use in College Health Centers: A Review. J. Am. Coll. Health.

[49] ESaewyc M., Konishi C., Rose H.A., Homma Y. (2014). School-Based Strategies to Reduce Suicidal Ideation, Suicide Attempts, and Discrimination among Sexual Minority and Heterosexual Adolescents in Western Canada. Int. J. Child Youth Fam. Stud..

[50] Aboud F.E., Fenwick V. (1999). Exploring and Evaluating School-Based Interventions to Reduce Prejudice. J. Soc. Isssues.

[51] Devine P.G., Forscher P.S., Austin A.J., Cox W.T.L. (2012). Long-term reduction in implicit race bias: A prejudice habit-breaking intervention. J. Exp. Soc. Psychol..

